# Whole-genome Sequencing Association Analysis of Quantitative Platelet Traits in A Large Cohort of β-thalassemia

**DOI:** 10.1093/gpbjnl/qzae065

**Published:** 2024-09-27

**Authors:** Xingmin Wang, Qianqian Zhang, Xianming Chen, Yushan Huang, Wei Zhang, Liuhua Liao, Xinhua Zhang, Binbin Huang, Yueyan Huang, Yuhua Ye, Mengyang Song, Jinquan Lao, Juanjuan Chen, Xiaoqin Feng, Xingjiang Long, Zhixiang Liu, Weijian Zhu, Lian Yu, Chengwu Fan, Deguo Tang, Tianyu Zhong, Mingyan Fang, Caiyun Li, Chao Niu, Li Huang, Bin Lin, Xiaoyun Hua, Xin Jin, Zilin Li, Xiangmin Xu

**Affiliations:** Innovation Center for Diagnostics and Treatment of Thalassemia, Nanfang Hospital, Southern Medical University, Guangzhou 510515, China; Department of Medical Genetics, School of Basic Medical Sciences, Southern Medical University, Guangzhou 510515, China; Innovation Center for Diagnostics and Treatment of Thalassemia, Nanfang Hospital, Southern Medical University, Guangzhou 510515, China; Department of Medical Genetics, School of Basic Medical Sciences, Southern Medical University, Guangzhou 510515, China; Dongguan Maternal and Child Health Care Hospital, Postdoctoral Innovation Practice Base of Southern Medical University, Dongguan 523001, China; Innovation Center for Diagnostics and Treatment of Thalassemia, Nanfang Hospital, Southern Medical University, Guangzhou 510515, China; Department of Medical Genetics, School of Basic Medical Sciences, Southern Medical University, Guangzhou 510515, China; College of Life Sciences, University of Chinese Academy of Sciences, Beijing 100049, China; BGI Research, Shenzhen 518083, China; Innovation Center for Diagnostics and Treatment of Thalassemia, Nanfang Hospital, Southern Medical University, Guangzhou 510515, China; Department of Medical Genetics, School of Basic Medical Sciences, Southern Medical University, Guangzhou 510515, China; Department of Pediatrics, Huizhou Central People’s Hospital, Huizhou 516001, China; Department of Hematology, 923(rd) Hospital of the People’s Liberation Army, Nanning 530021, China; Department 1 of Internal Medicine, Sixth People’s Hospital of Nanning, Nanning 530022, China; Department of Pediatrics, Affiliated Hospital of Youjiang Medical University for Nationalities, Baise 533000, China; Innovation Center for Diagnostics and Treatment of Thalassemia, Nanfang Hospital, Southern Medical University, Guangzhou 510515, China; Department of Medical Genetics, School of Basic Medical Sciences, Southern Medical University, Guangzhou 510515, China; Innovation Center for Diagnostics and Treatment of Thalassemia, Nanfang Hospital, Southern Medical University, Guangzhou 510515, China; Department of Medical Genetics, School of Basic Medical Sciences, Southern Medical University, Guangzhou 510515, China; Department of Pediatrics, Liuzhou Worker’s Hospital, Liuzhou 545005, China; Department of Pediatrics, Shenzhen Second People’s Hospital, The First Affiliated Hospital of Shenzhen University, Shenzhen 518035, China; Department of Pediatrics, Nanfang Hospital, Southern Medical University, Guangzhou 510515, China; Department of Pediatrics, Liuzhou People’s Hospital, Liuzhou 545001, China; Department of Health Care, Heyuan Maternal and Child Health Care Hospital, Heyuan 517000, China; Department of Hematology and Oncology, Zhuhai People’s Hospital, The Third Affiliated Hospital, Jinan University Medical College, Zhuhai 519000, China; Department of Hematology and Rheumatology, Longyan First Hospital, Affiliated to Fujian Medical University, Longyan 364000, China; Department of pediatrics, Second People’s Hospital of Guilin, Guilin 541001, China; Maternal and Child Health Hospital of Yongzhou, Yongzhou 425000, China; Department of Clinical Laboratory, The First Affiliated Hospital of Gannan Medical University, Ganzhou 341000, China; BGI Research, Shenzhen 518083, China; Center of Prenatal Diagnosis, Chenzhou No.1 People’s Hospital, Chenzhou 423000, China; Innovation Center for Diagnostics and Treatment of Thalassemia, Nanfang Hospital, Southern Medical University, Guangzhou 510515, China; Department of Medical Genetics, School of Basic Medical Sciences, Southern Medical University, Guangzhou 510515, China; Innovation Center for Diagnostics and Treatment of Thalassemia, Nanfang Hospital, Southern Medical University, Guangzhou 510515, China; Department of Medical Genetics, School of Basic Medical Sciences, Southern Medical University, Guangzhou 510515, China; Guangzhou Huayin Healthcare Group Co., Ltd., Guangzhou 510663, China; Guangzhou Huayin Healthcare Group Co., Ltd., Guangzhou 510663, China; BGI Research, Shenzhen 518083, China; School of Mathematics and Statistics and KLAS, Northeast Normal University, Changchun 130024, China; Innovation Center for Diagnostics and Treatment of Thalassemia, Nanfang Hospital, Southern Medical University, Guangzhou 510515, China; Department of Medical Genetics, School of Basic Medical Sciences, Southern Medical University, Guangzhou 510515, China

**Keywords:** β-thalassemia, Phenotypic heterogeneity, Platelet count, Mean platelet volume, Whole-genome sequencing analysis

## Abstract

Platelets act as a crucial indicator for monitoring hypercoagulability and thrombosis and a key target for pharmacological intervention. Genotype–phenotype association studies have confirmed that platelet traits are quantitatively regulated by multiple genes. However, there is currently a lack of genetic studies on the heterogeneity of platelet traits in β-thalassemia under a hypercoagulable state. Here, we studied the phenotypic heterogeneity of platelet count (PLT) and mean platelet volume (MPV) in a cohort of 1020 β-thalassemia patients. We further performed a functionally informed whole-genome sequencing (WGS) association analysis of common variants and rare variants for PLT and MPV in 916 patients through integrative analysis of WGS data and functional annotation data. Extreme phenotypic heterogeneity of platelet traits was observed in β-thalassemia patients. Additionally, the common variant-based gene-level analysis identified *RNF144B* as a novel gene associated with MPV. The rare variant analysis identified several novel associations in both coding and noncoding regions, including missense rare variants of *PPP2R5C* associated with PLT and missense rare variants of *TSSK1B* associated with MPV. In conclusion, this comprehensive and systematic whole-genome scan of platelet traits in the β-thalassemia cohort reveals the specific genetic regulation of platelet traits in the context of β-thalassemia, providing potential targets for intervention.

## Graphical abstract

**Graphical abstract:**
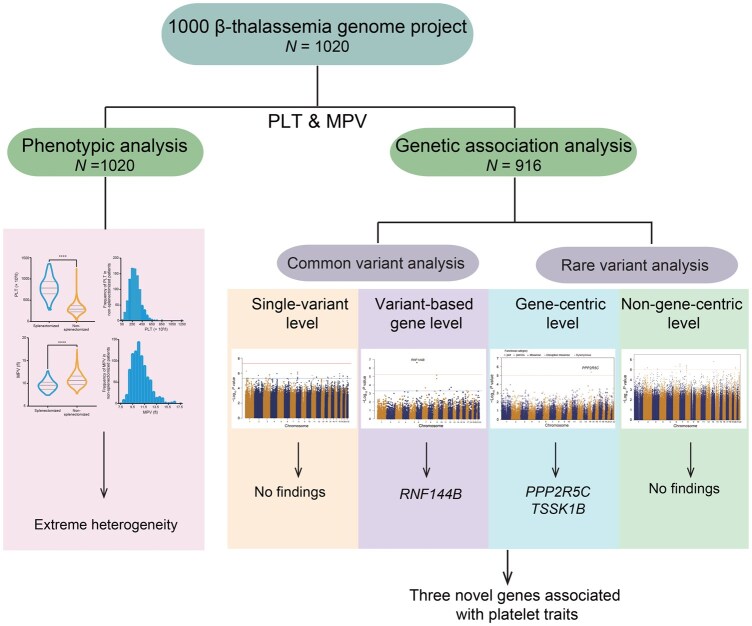

## Introduction

Platelets are enucleated cellular fragments derived from megakaryocytes. They play a crucial role in the pathogenesis of hemostasis and thrombosis, as well as in several biological processes including wound healing, immune and inflammatory response, vascular integrity, and tumor metastasis [1–3]. Deviations from normal platelet parameters can indicate certain disease states [4]. Among these parameters, platelet count (PLT) and mean platelet volume (MPV) are commonly measured to assess platelet characteristics in clinical diagnosis, and they exhibit a negative correlation with each other [5]. Abnormal platelet levels can lead to severe bleeding disorders, thrombotic diseases, and cardiovascular diseases [6], which are often managed with aspirin and dual antiplatelet therapy [7].

β-thalassemia is one of the most prevalent monogenic inherited diseases worldwide and has a geographical distribution overlapping with historical malaria endemicity [8]. It is caused by mutations in the gene encoding the β-globin chain of hemoglobin, leading to an imbalance in α/β-globin chain ratio, ineffective erythropoiesis, chronic hemolysis, hypercoagulable state, and increased intestinal iron absorption [8]. Severe forms of β-thalassemia manifest various complications, posing a series of management challenges due to significant clinical heterogeneity and the lack of relevant prognostic markers. Thromboembolic event is one of the common complications in β-thalassemia patients due to their long-term hypercoagulability, with splenectomy and elevated platelet levels being the potential risk factors [9]. In β-thalassemia, low PLT is commonly associated with hypersplenism, while high PLT may be susceptible to thromboembolism [10,11]. Increased MPV typically signals platelet activation and could indicate a compensatory response to peripheral platelet destruction or consumption. Conversely, low MPV might reflect bone marrow dysfunction or the effects of splenomegaly on platelet production [12]. Therefore, regular monitoring of PLT and MPV is essential for the effective clinical management of β-thalassemia. However, there is a lack of research analyzing the distribution of PLT and MPV among β-thalassemia patients.

Genome-wide association studies (GWAS) have identified thousands of platelet-related genetic variants, explaining a considerable portion of platelet traits. The heritability of PLT and MPV was estimated to be 25%–87% and 40%–50%, respectively, suggesting a significant genetic influence on platelet traits [4,6,13,14]. Previous studies have utilized PLT-associated genetic variations to investigate the involvement of relevant genes in regulating acute respiratory distress syndrome survival rate, uncovering potential therapeutic targets [15–17]. With the development of high-throughput sequencing, high-coverage whole-genome sequencing (WGS) is becoming more available, facilitating comprehensive genetic information discovery, particularly for rare variants [minor allele frequency (MAF) < 1%]. Rare variants constitute the majority proportion of variants in the human genome [18], and recent studies have identified that rare variants cause monogenic diseases in several cases [19,20]. However, a research gap exists in understanding genetic variations associated with platelet traits in β-thalassemia patients.

Here, we studied the heterogeneity of PLT and MPV in 1020 β-thalassemia patients, using a comprehensive clinical phenotype. Additionally, we explored the full allelic spectrum associated with PLT and MPV in 916 non-splenectomized β-thalassemia patients through integrative analysis of WGS data and functional annotation data. Our analyses encompassed functionally informed WGS association analysis of 33.43 million common and rare variants, including single variant, gene-level, gene-centric, and non-gene-centric analyses. We subsequently performed follow-up conditional analysis to identify association signals independent of known single variant associations indexed in the GWAS Catalog [21]. The findings of our study elucidated significant heterogeneity in platelet traits among β-thalassemia cases, contributing new insights into the genetic architecture of platelet traits within this condition. These insights deepen our understanding of the pathophysiological mechanisms underlying β-thalassemia and spotlight potential targets for innovative therapeutic interventions.

## Results

### Overview

**Figure 1:**
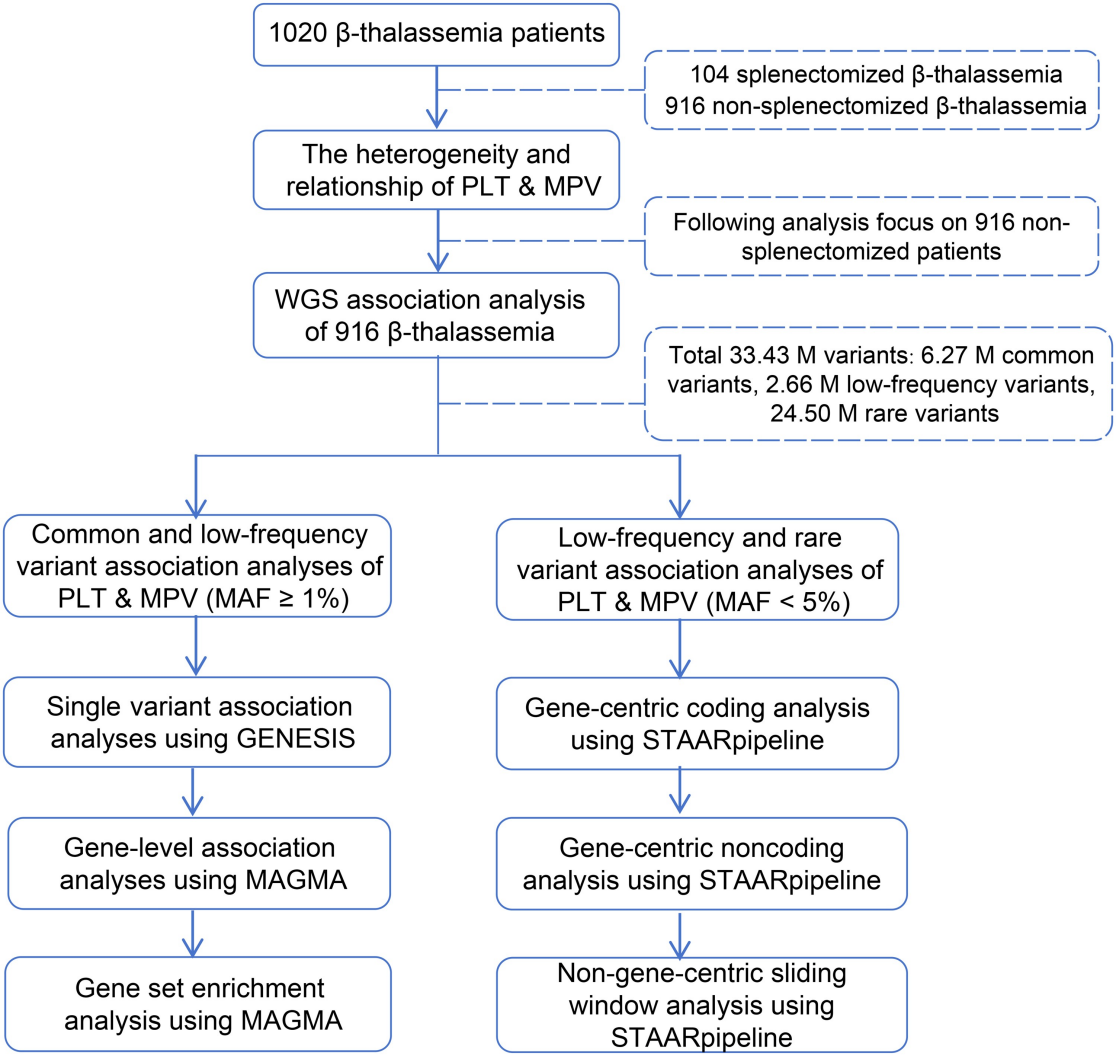
The workflow of our study The analyses of PLT and MPV heterogeneity and their relationship were conducted on a cohort of 1020 β-thalassemia patients. PLT and MPV exhibited significant phenotypic heterogeneity, particularly among 916 non-splenectomized β-thalassemia patients. To explore the genetic factors underlying this phenotypic heterogeneity, association studies were performed on 33.43 M variants from 916 patients to identify genes and genomic regions associated with PLT and MPV. Single variant and gene-level association analyses were performed using GENESIS and MAGMA, respectively, with common and low-frequency variants (MAF ≥ 1%). Genome-wide low-frequency and rare variant (MAF < 5%) gene-centric and non-gene-centric aggregate tests were performed using STAARpipeline. PLT, platelet count; MPV, mean platelet volume; MAF, minor allele frequency; M, million; MAGMA, Multi-marker Analysis of GenoMic Annotation.

We conducted a comprehensive analysis of the phenotypic heterogeneity of two platelet traits, PLT and MPV, and evaluated the impact of splenectomy on these traits in a cohort of 1020 β-thalassemia patients. Subsequently, we investigated the genetic factors contributing to the phenotypic heterogeneity of PLT and MPV in β-thalassemia utilizing WGS data from 916 non-splenectomized β-thalassemia patients. For common and low-frequency variants (MAF ≥ 1%), we performed single variant analysis on 8.93 million individual autosomal variants. Additionally, we conducted gene-level association studies and gene set enrichment analyses to augment the insights derived from the single variant association analyses. For low-frequency and rare variants (MAF < 5%), we performed variant set analysis on 27.16 million autosomal variants, employing functionally informed association analysis. This comprehensive approach encompassed both gene-centric and non-gene-centric analyses using various coding and noncoding masks defined by categorical functional annotations. Furthermore, we integrated multiple quantitative variant functional annotation scores representing multi-aspect variant biological functions for each variant set, thereby enhancing the analytical robustness and power. A schematic overview of our study is displayed in **Figure 1**.

### Phenotypic heterogeneity of PLT and MPV in β-thalassemia

#### Phenotypic heterogeneity analysis

**Figure 2:**
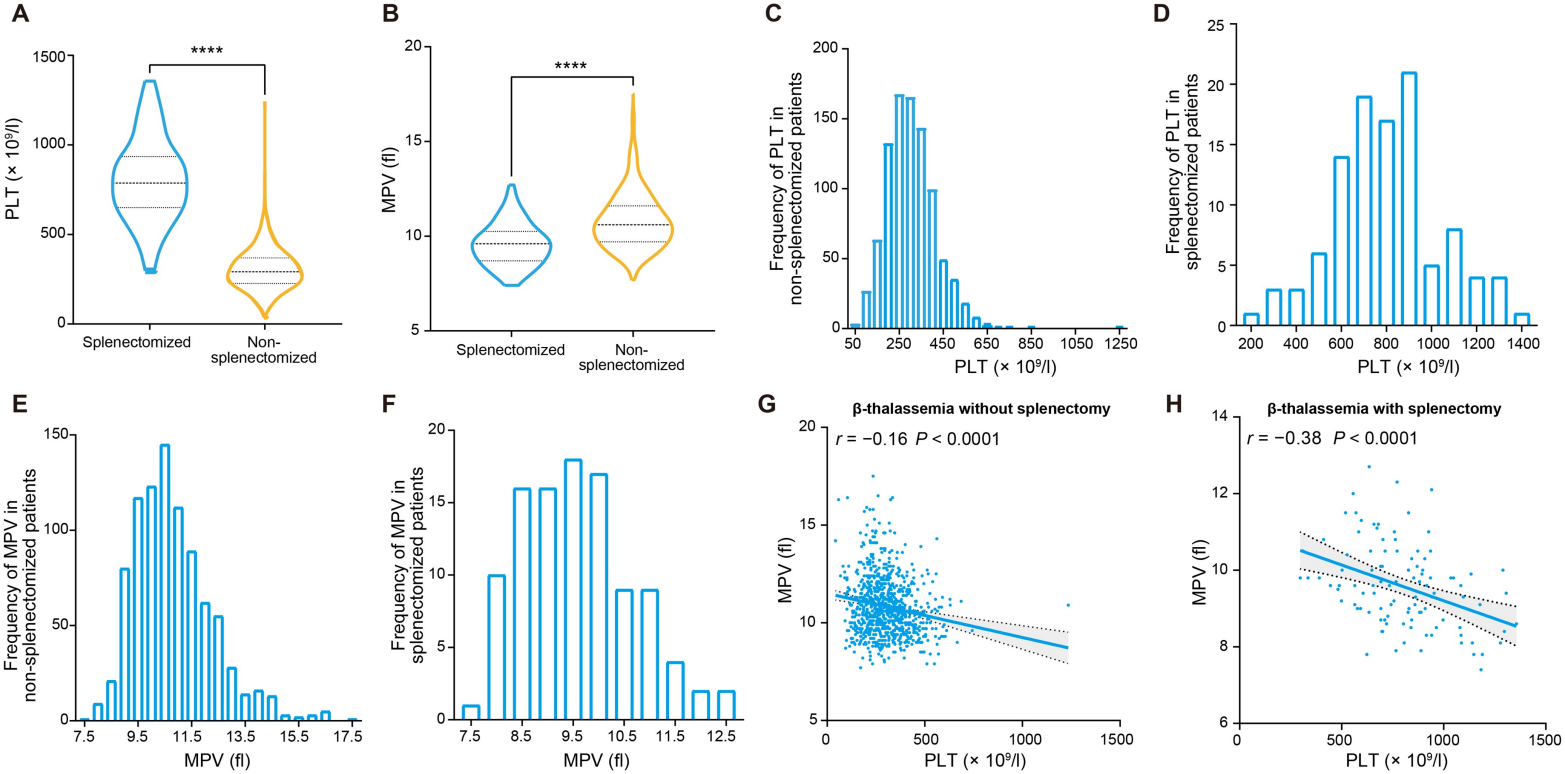
Analysis of PLT and MPV in splenectomized and non-splenectomized **β**-thalassemia patients **A**. Statistical analysis of PLT between splenectomized and non-splenectomized β-thalassemia patients. **B**. Statistical analysis of MPV between splenectomized and non-splenectomized β-thalassemia patients. **C**. Distribution of PLT in β-thalassemia patients without splenectomy, showing considerable phenotypic heterogeneity in PLT. **D**. Distribution of PLT in β-thalassemia patients with splenectomy, showing considerable phenotypic heterogeneity in PLT. **E**. Distribution of MPV in β-thalassemia patients without splenectomy, showing considerable phenotypic heterogeneity in MPV. **F**. Distribution of MPV in β-thalassemia patients with splenectomy, showing considerable phenotypic heterogeneity in MPV. **G**. Relationship between PLT and MPV in non-splenectomized β-thalassemia patients, showing a significant negative correlation. **H**. Relationship between PLT and MPV in splenectomized β-thalassemia patients. In (A and B), significant difference was determined by Spearman rank correlation analysis (****, *P* ≤ 0.0001).

We conducted a thorough analysis of the clinical characteristics of 1020 β-thalassemia patients recruited from southern China (**Table 1**). Our findings reveal that PLT levels are significantly elevated in splenectomized β-thalassemia patients compared to non-splenectomized patients, whereas MPV levels are notably lower in splenectomized patients (**Figure 2**A and B). These results suggest a considerable impact of splenectomy on both PLT and MPV in β-thalassemia patients. Furthermore, our investigation into the frequency distribution of PLT and MPV among splenectomized and non-splenectomized β-thalassemia patients demonstrates substantial phenotypic heterogeneity in both groups (Figure 2C–F). While splenectomy exerts a discernible effect on these platelet traits, significant phenotypic variability persists across the entire cohort, particularly among non-splenectomized patients. Consequently, our subsequent analysis was dedicated to elucidating potential genetic variations influencing PLT and MPV traits in non-splenectomized β-thalassemia patients.

**Table 1:** Clinical characteristics of 1020 β-thalassemia patients in this study

Characteristic	Patients without splenectomy (*N* = 916)	Patients with splenectomy (*N* = 104)	*P* value
Sex (male:female)	516:400	58:46	0.913
Age (month; mean ± SD)	123.85 ± 55.76	192.68 ± 89.75	< 0.001
*HBB* genotype	635:254:9:12:4:2	65:36:1:2:0:0	0.680
Clinical classification (TM:TI)	704:212	71:33	0.052
MPV (fl; mean ± SD)	10.82 ± 1.48	9.57 ± 1.11	< 0.001
PLT (× 10^9^/l; mean ± SD)	304.98 ± 113.17	804.42 ± 232.33	< 0.001

*Note: HBB* genotype includes β^0^/β^0^, β^0^/β^+^, β^+^/β^+^, β^0^/HPEH, β^0^/N, and β^0^/N + α-duplication. For clinical classification, the transfusion-free survival time less than 24 months is defined as TM, and the transfusion-free survival time more than or equal to 24 months is defined as TI. TM, thalassemia major; TI, thalassemia minor; PLT, platelet count; MPV, mean platelet volume; SD, standard deviation.

#### Clinical and genetic relationship analyses

We performed a relationship analysis of PLT and MPV in non-splenectomized β-thalassemia patients (*N* = 899, with 17 patients lacking MPV values) and splenectomized β-thalassemia patients (*N* = 104). In both patient groups, we observed a negative correlation between PLT and MPV, with a Spearman correlation coefficient of −0.16 (*P* < 0.001) and −0.38 (*P* < 0.001), respectively (Figure 2G and H). Furthermore, we calculated a genetic correlation coefficient of −0.29 [standard error (SE) = 0.49] between PLT and MPV in non-splenectomized patients. The heritability estimated for PLT and MPV was 0.32 (SE = 0.25) and 0.38 (SE = 0.25), respectively. These findings collectively demonstrate a consistent negative relationship between PLT and MPV across both clinical and genetic aspects.

### Common variant analyses unveiled associated signals and enriched functions

#### Single variant analysis

**Figure 3:**
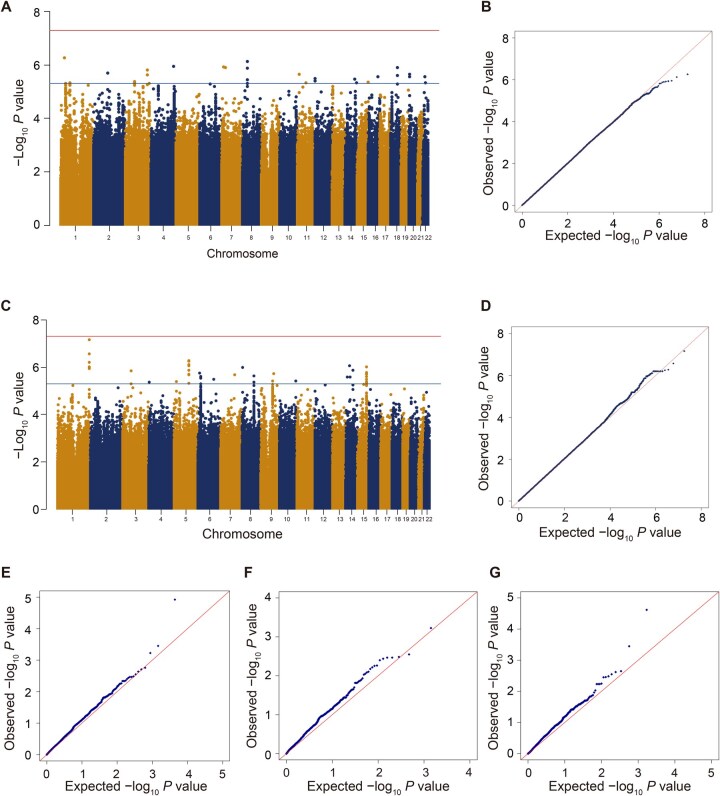
Results of PLT and MPV single variant association studies using GENESIS **A**. Manhattan plot of PLT single variant association study. Red line represents significant *P* = 5.00E−08, and blue line represents suggestive *P* = 5.00E−06. **B**. QQ plot of PLT single variant association study. **C**. Manhattan plot of MPV single variant association study. Red line represents significant *P* = 5.00E−08, and blue line represents suggestive *P* = 5.00E−06. **D**. QQ plot of MPV single variant association study. **E**. QQ plot of 2189 shared variants, known to be the significant loci associated with PLT across all populations and β-thalassemia cohort in this study. **F**. QQ plot of 708 shared variants, known to be the significant loci associated with PLT in East Asian populations and β-thalassemia cohort in this study. **G**. QQ plot of 854 shared variants, known to be the significant loci associated with MPV across all populations and β-thalassemia cohort in this study. QQ, quantile–quantile.

We conducted single variant association studies of PLT and MPV, analyzing 8.93 million individual autosomal variants (MAF ≥ 1%) (**Figure 3**A–G). The genomic inflation factors were 1.008 for PLT and 1.012 for MPV, respectively, indicating adequate control for population stratification (Figure 3B and D). While no variants passed genome-wide significance (*P* < 5.00E−08), multiple variants surpassed the suggestively significant threshold (*P* < 5.00E−06) (Table S1). Notably, all these variants were identified as novel after conditional analysis, conditioning on known PLT-associated or MPV-associated variants, respectively (Table S2). Among these variants, rs78326374 emerged as the top variant in the PLT association test (*P* = 5.51E−07), located in the intronic region of *EPB41* (Figure S1A and D; **Table 2**). Previous studies have implicated the *OPRD1–EPB41* gene region in significant associations with platelet and red blood cell (RBC) parameters [14,22,23]. Known platelet-associated variants in this region exhibited *P* values with no statistical difference and weak linkage disequilibrium (LD) (*r*^2^ < 0.1) with rs78326374 in our cohort. Additionally, rs144319949 emerged as the second top variant in the PLT association analysis (*P* = 7.44E−07), located in a novel region associated with platelet traits (Figure S1B and E). This variant exhibited low frequency in the East Asian population and was extremely rare in other ethnic populations (Table 2). In the MPV single variant association study, rs117379094 was the top variant (*P* = 6.82E−08) (Figure S1C and F; Table 2), located in the intergenic region of *CHRM3*. A previous study has identified that rs6677208 located in the intron of *CHRM3* is associated with PLT [24], but it was not observed in our cohort as well as the East Asian population [25].

**Table 2:** Top variants from single variant association studies of PLT and MPV

Trait	rs ID	Chr	Pos (bp)	MAF	Ref	Alt	Gene	Function	PVE	Est	Est.SE	Score.pval	EAS	SAS	AFR	EUR
PLT	rs78326374	1	28968982	0.44	C	A	*EPB41*	Intronic	0.028	−23.55	4.70	5.51E−07	0.39	0.25	0.24	0.12
PLT	rs144319949	8	34545785	0.03	C	A	LINC01288	Intergenic	0.027	71.47	14.44	7.44E−07	0.02	0	NA	NA
MPV	rs117379094	1	238497694	0.07	T	C	LINC01139; *CHRM3*	Intergenic	0.033	−0.72	0.13	6.82E−08	0.07	0.05	0	0.03

*Note*: Chr, chromosome; Pos, position; Ref, reference; Alt, alteration; MAF, minor allele frequency; PVE, phenotypic variance explained; Est, effect size for each effect allele; Est.SE, standard error of effect size estimate; Score.pval, *P* value of score test in this study; EAS, 1000 Genomes East Asian population frequency; SAS, 1000 Genomes South Asian population frequency; AFR, 1000 Genomes African population frequency; EUR, 1000 Genomes European population frequency; NA, not available.

#### Gene-level analysis

**Figure 4:**
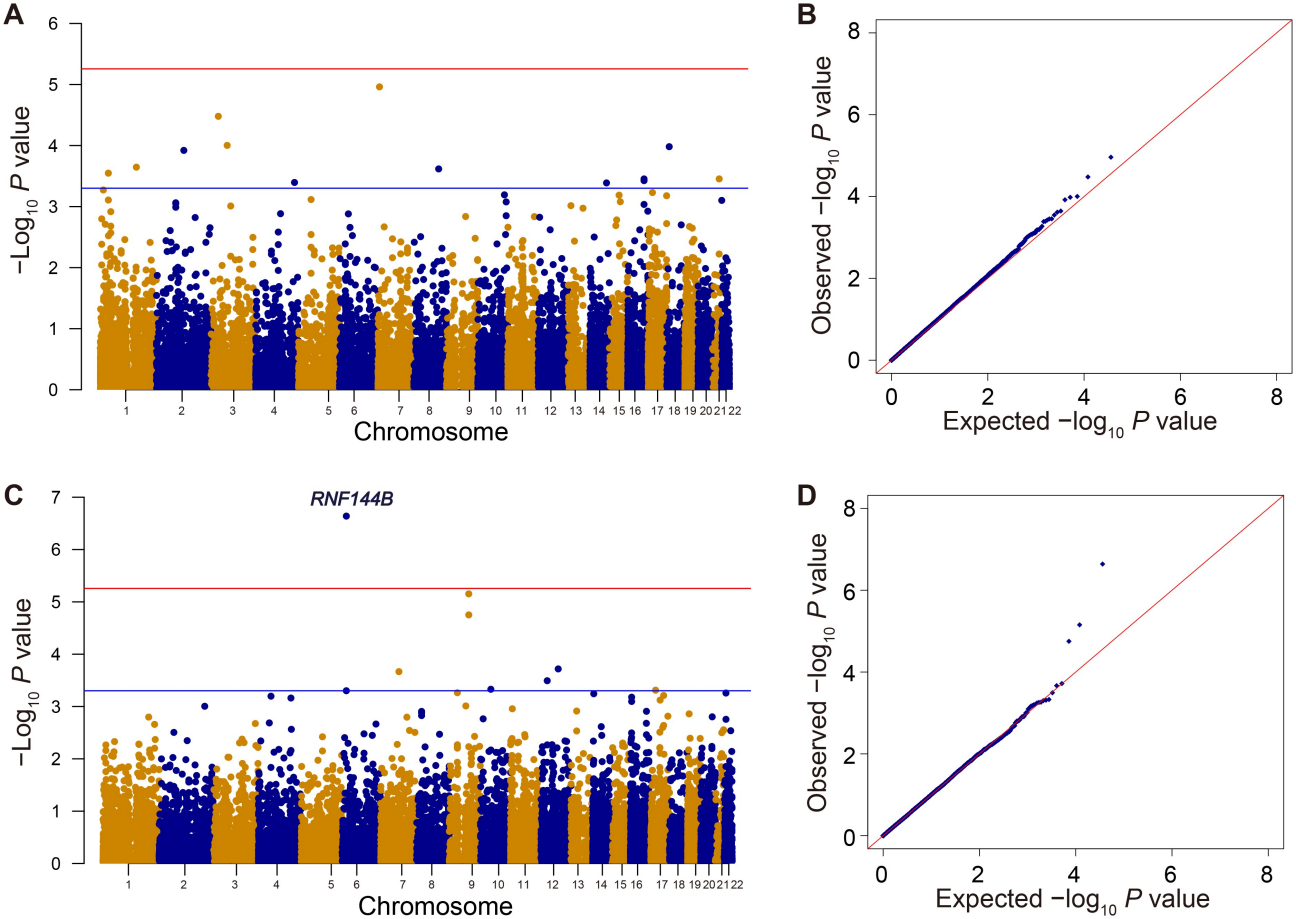
Results of PLT and MPV gene-level association studies using MAGMA **A**. Manhattan plot of PLT gene-level association study. Red line represents Bonferroni-corrected threshold *P* = 2.77E−06, and blue line represents suggestive *P* = 5.00E−04. **B**. QQ plot of PLT gene-level association study. **C**. Manhattan plot of MPV gene-level association study. *RNF144B* is the gene significantly associated with MPV. Red line represents Bonferroni-corrected threshold *P* = 2.77E−06, and blue line represents suggestive *P* = 5.00E−04. **D**. QQ plot of MPV gene-level association study.

We conducted gene-level association studies of PLT and MPV, analyzing 18,052 protein-coding genes with the single variant summary statistics (**Figure 4**A–D; Table S3). The genome-wide significant threshold for gene-level analyses was defined at a Bonferroni-corrected *P* value of 5.54E−06 (*P* = 0.1/18,052). In the PLT gene-level analysis, no genes surpassed the genome-wide significant threshold, and the genomic inflation factor was 1.070. However, in the MPV gene-level analysis, *RNF144B* was identified as a novel gene associated with MPV (*P* = 2.30E−07) with the genomic inflation factor as 1.015.

#### Functional enrichment analysis

We performed gene set enrichment analysis (GSEA) of PLT and MPV to assess their impact on biological functions and possible diseases. While no gene set achieved the Bonferroni-corrected significance threshold (*P* = 6.26E−06, corresponding to *P* = 0.1/15,978), the top items highlighted several platelet-related functions (Table S4). In PLT GSEA, enriched functions included positive regulation of receptor-mediated endocytosis (*P* = 6.76E−04), deep venous thrombosis (*P* = 8.00E−04), and platelet alpha granule (*P* = 1.76E−03). Similarly, the MPV GSEA revealed enrichment in functions such as ATP-dependent protein folding chaperone (*P* = 2.89E−04), chemokine binding (*P* = 5.33E−04), cell–cell adhesion via plasma membrane adhesion molecules (*P* = 8.31E−04), and phagocytosis (*P* = 1.16E−03). These findings underscore the biological significance of platelets and their close association with inflammatory processes.

Given the pivotal role of platelets in inflammation, we conducted further investigations into the correlation between PLT or MPV and inflammatory cells (including counts of white blood cells, monocytes, lymphocytes, neutrophils, eosinophils, and basophils) within our β-thalassemia cohort. Our analysis revealed a significant positive correlation between PLT and all six inflammatory cell counts (Figure S2A–F), while MPV exhibited a significant negative correlation with them (Figure S2G–L). These consistent findings aligned with underlying biological processes and underscored the contribution of platelets to the inflammatory response.

### Rare variant analyses identified novel associated genes

**Figure 5:**
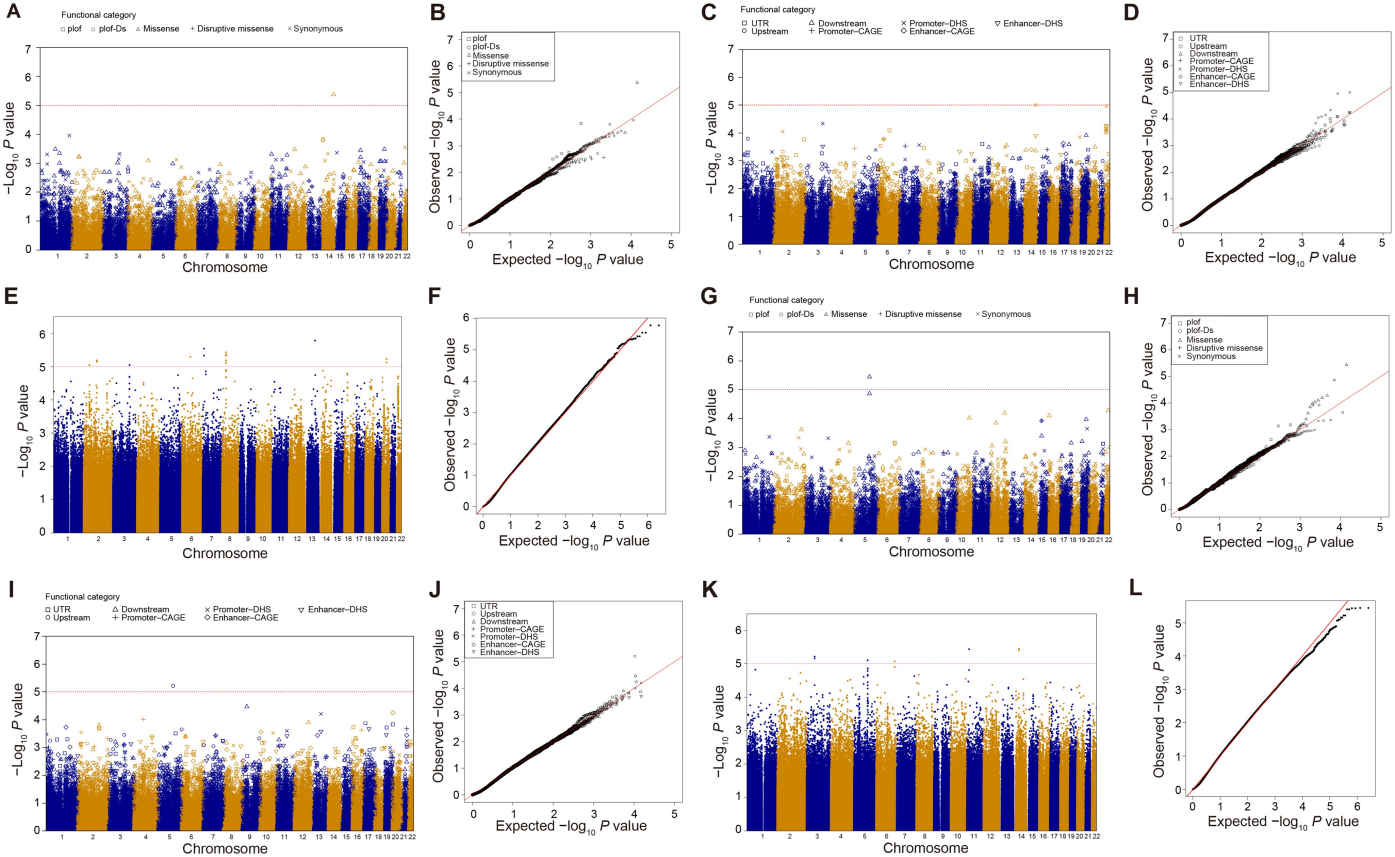
Results of low-frequency and rare variant association studies for PLT and MPV using STAARpipeline **A**. Manhattan plot for unconditional PLT gene-centric coding analysis of protein-coding genes. Red horizontal line indicates the suggestive significance threshold STAAR-O *P* = 1.00E−05. **B**. QQ plot for unconditional PLT gene-centric coding analysis of protein-coding genes. **C**. Manhattan plot for unconditional PLT gene-centric noncoding analysis of protein-coding genes. Red horizontal line indicates the suggestive significance threshold STAAR-O *P* = 1.00E−05. **D**. QQ plot for unconditional PLT gene-centric noncoding analysis of protein-coding genes. **E**. Manhattan plot for PLT 4-kb sliding window analysis with 2-kb skip length. Red horizontal line indicates the suggestive significance threshold STAAR-O *P* = 1.00E−05. **F**. QQ plot for PLT 4-kb sliding window analysis with 2-kb skip length. **G**. Manhattan plot for unconditional MPV gene-centric coding analysis of protein-coding genes. Red horizontal line indicates the suggestive significance threshold STAAR-O *P* = 1.00E−05. **H**. QQ plot for unconditional MPV gene-centric coding analysis of protein-coding genes. **I**. Manhattan plot for unconditional MPV gene-centric noncoding analysis of protein-coding genes. Red horizontal line indicates the suggestive significance threshold STAAR-O *P* = 1.00E−05. **J**. QQ plot for unconditional MPV gene-centric noncoding analysis of protein-coding genes. **K**. Manhattan plot for MPV 4-kb sliding window analysis with 2-kb skip length. Red horizontal line indicates the suggestive significance threshold STAAR-O *P* = 1.00E−05. **L**. QQ plot for MPV 4-kb sliding window analysis with 2-kb skip length. plof, putative loss-of-function; plof-Ds, putative loss-of-function or disruptive missense; DHS, DNase I hypersensitive site; CAGE, cap analysis of gene expression; promoter–CAGE, promoter variants within CAGE sites; promoter–DHS, promoter variants within DHSs; enhancer–CAGE, enhancer variants within CAGE sites; enhancer–DHS, enhancer variants within DHSs; UTR, untranslated region.

We evaluated the associations of aggregated low-frequency and rare autosomal variants (MAF < 5%). In the PLT gene-centric coding analysis, we identified a genome-wide significant gene, *PPP2R5C*, harboring missense rare variants (Bonferroni-corrected significant *P* = 0.1/20,000 = 5.00E−06, STAAR-O *P* = 4.15E−06) (**Figure 5**A and B; **Table 3**). In the PLT gene-centric noncoding analysis, promoter variants within DNase I hypersensitive sites (DHSs) in *PPP2R5C* were identified as a potentially suggestive significant region (STAAR-O *P* = 9.80E−06) (Figure 5C and D; Table 3). Additionally, in the PLT non-gene-centric analysis (Figure 5E and F; **Table 4**), we identified 16 suggestively associated windows. Among these, chr13:83284912–83288911 (intergenic region of AL445255.1 and *RNU6-67P*) was the top suggestive region in the PLT sliding window analysis (STAAR-O *P* = 1.67E−06). Importantly, all these associations remained significant after adjustment for PLT-specific variants using conditional analysis (Tables 3 and 4).

**Table 3:** Results of gene-centric coding and noncoding analyses for PLT and MPV

Type	Trait	Gene	Chr	Category	No. of SNVs	STAAR-O *P* (unconditional)	STAAR-O *P* (conditional)	Variants adjusted (conditional)
Coding	PLT	*PPP2R5C*	14	Missense	5	4.15E−06	4.15E−06	NA
Coding	MPV	*TSSK1B*	5	Missense	11	3.66E−06	3.66E−06	NA
Noncoding	PLT	*PPP2R5C*	14	Promoter–DHS	18	9.80E−06	9.80E−06	NA
Noncoding	MPV	*MCC*	5	Upstream	11	6.22E−06	6.22E−06	NA

*Note*: SNV, single nucleotide variant; DHS, DNase I hypersensitive site.

**Table 4:** Results of 4-kb sliding window analyses for PLT and MPV

Trait	Chr	Start (bp)	End (bp)	Gene	No. of SNVs	STAAR-O *P* (unconditional)	STAAR-O *P* (conditional)	Variants adjusted (conditional)
PLT	13	83284912	83288911	Intergenic (AL445255.1, *RNU6-67P*)	39	1.67E−06	1.67E−06	NA
PLT	7	12241256	12245255	Intergenic (*TMEM106B*, *VWDE*)	29	2.87E−06	2.87E−06	NA
PLT	8	34483807	34487806	Intergenic (AC090993.1, LINC01288)	34	3.74E−06	3.74E−06	NA
PLT	8	34541807	34545806	Intergenic (AC090993.1, LINC01288)	10	4.40E−06	4.40E−06	NA
PLT	8	34543807	34547806	Intergenic (AC090993.1, LINC01288)	21	4.66E−06	4.66E−06	NA
PLT	7	12243256	12247255	Intergenic (*TMEM106B*, *VWDE*)	40	4.67E−06	4.67E−06	NA
PLT	6	69843582	69847581	Intronic (*LMBRD1*)	42	5.11E−06	5.11E−06	NA
PLT	20	38382454	38386453	ncRNA_intronic (AL391095.2)	36	5.84E−06	5.84E−06	NA
PLT	8	34401807	34405806	Intergenic (AC090993.1, LINC01288)	32	6.40E−06	6.40E−06	NA
PLT	2	110792535	110796534	Intronic (*ACOXL*)	39	6.50E−06	6.50E−06	NA
PLT	8	34399807	34403806	Intergenic (AC090993.1, LINC01288)	28	6.58E−06	6.58E−06	NA
PLT	2	110794535	110798534	Intronic (*ACOXL*)	41	7.02E−06	7.02E−06	NA
PLT	20	38380454	38384453	Intergenic (AL391095.2, AL391095.1)	30	7.46E−06	7.46E−06	NA
PLT	8	34363807	34367806	Intergenic (AC090993.1, LINC01288)	26	7.81E−06	7.81E−06	NA
PLT	3	136901859	136905858	Intronic (*NCK1*)	42	8.82E−06	8.82E−06	NA
PLT	2	48836535	48840534	ncRNA_intronic (AC009975.1)	54	8.98E−06	8.98E−06	NA
MPV	14	44232771	44236770	ncRNA_intronic (LINC02307)	26	3.57E−06	3.57E−06	NA
MPV	11	17996581	18000580	Intronic (*SERGEF*)	25	3.65E−06	3.65E−06	NA
MPV	14	44230771	44234770	ncRNA_intronic (LINC02307)	35	3.93E−06	3.93E−06	NA
MPV	3	65805859	65809858	Intronic (*MAGI1*)	39	5.99E−06	5.99E−06	NA
MPV	3	65807859	65811858	Intronic (*MAGI1*)	45	6.90E−06	6.90E−06	NA
MPV	5	113387917	113391916	ncRNA_intronic (AC079465.1)	42	7.93E−06	7.93E−06	NA
MPV	6	153995582	153999581	Intergenic (*RNU6-896P*, *OPRM1*)	29	8.55E−06	8.55E−06	NA

In the MPV gene-centric coding analysis, we discovered a genome-wide significant gene, *TSSK1B*, harboring missense rare variants (Bonferroni-corrected significant *P* = 0.1/20,000 = 5.00E−06, STAAR-O *P* = 3.66E−06) (Figure 5G and H; Table 3). Moreover, in the MPV gene-centric noncoding analysis, upstream rare variants in *MCC* were identified as a suggestively significant region (STAAR-O *P* = 6.22E−06) (Figure 5I and J; Table 3). Additionally, the MPV non-gene-centric analysis revealed seven suggestively associated windows (Figure 5K and L; Table 4). Among them, chr14:44232771–44236770 (intronic region of LINC02307) emerged as the top suggestive region in the MPV sliding window analysis (STAAR-O *P* = 3.57E−06). Importantly, these associations remained significant after adjusting for MPV-specific variants (Tables 3 and 4).

To explore the role of protein-truncating variants (PTVs), we performed an additional analysis across protein-coding genes, including two masks of PTV and PTV + disruptive missense variant (PTV + D). None of the associations of the masks of PTV and PTV + D rare variants achieved genome-wide significance at the level of 5.00E−06 (Figure S3).

## Discussion

We conducted a comprehensive analysis of phenotypic heterogeneity in PLT and MPV within a large β-thalassemia cohort, aiming to identify genetic loci associated with these platelet traits. Notably, we observed a significant influence of splenectomy on both PLT and MPV in β-thalassemia patients, underscoring the importance of considering clinical factors in genetic studies. By leveraging deep-coverage WGS data, we conducted association analyses for PLT and MPV using all genetic variants. Considering racial disparities and the unique disease background of β-thalassemia, our results revealed shared and distinctive genetic findings compared to previous studies. Notably, *PPP2R5C* was identified as significantly associated with PLT, while *TSSK1B* and *RNF144B* were significantly associated with MPV. Additionally, enrichment analysis unveiled multiple signals in inflammation-related pathways, such as phagocytosis and chemotaxis, aligning with the correlation between platelet traits and leukocyte-related parameters. These findings underscore the pivotal role of platelets in orchestrating and modulating inflammatory responses through interactions with leukocytes, shedding light on potential mechanisms underlying platelet-mediated inflammation.

Our cohort, comprising 1020 β-thalassemia patients from southern China, represents the largest dataset of its kind with both comprehensive clinical phenotyping and WGS information. The cohort mainly consisted of patients with thalassemia major and intermedia, with approximately 90% being non-splenectomized. Our clinical findings highlight the significant impact of splenectomy on platelet traits, aligning with previous research [5], and confirm the negative correlation between PLT and MPV in β-thalassemia. Notably, our observations reveal considerable phenotypic heterogeneity in PLT and MPV among β-thalassemia patients, especially in those without splenectomy. Additionally, utilizing WGS data, we have identified a negative genetic correlation between PLT and MPV, suggesting the presence of shared regulatory loci exerting opposing effects on these traits.

We conducted association studies using 33,430,783 autosomal variants, including common, low-frequency, and rare variants. In the absence of an independent validation cohort, we sought to validate our findings and ascertain the specificity of our observations within disease cohorts by examining variants previously reported to be associated with PLT or MPV from the GWAS Catalog [21] (Table S2). There are 3093 loci associated with PLT reported in the GWAS Catalog [21] (all association files dated February 20, 2024), of which 2189 variants were observed in our study. Among these 2189 variants, 708 variants were observed in East Asian populations [21]. There are 1380 loci associated with MPV reported in the GWAS Catalog [21] (all association files dated February 20, 2024), of which 854 variants were observed in our study. We constructed quantile–quantile (QQ) plots by extracting the *P* values of these loci from our single variant summary statistics (Figure 3E–G). Our plots show that these loci are probably subject to natural selection and participate in influencing PLT or MPV. While these loci were validated in our cohort, the limited sample size prevented them from reaching statistical significance (*P* = 5.00E−08). Nonetheless, these results underscore the similarities and specificity between our cohort and previous datasets. Importantly, our study, conducted in the context of β-thalassemia, uncovered novel associated variants, genes, or regions, distinct from those identified in studies focusing on healthy individuals.

Through comprehensive WGS association analysis of all variants, we have identified three genes significantly associated at the genome-wide level with PLT or MPV including *PPP2R5C*, *TSSK1B*, and *RNF144B*. Additionally, several genes have shown suggestive associations with platelet traits including *EPB41*, *SMOX*, and *CHRM3*. Among them, *PPP2R5C*, *RNF144B*, and *TSSK1B* are newly discovered genes associated with platelet traits, while *EPB41* [14,22,23] and *CHRM3* [24] have been known to be associated with platelets. The protein encoded by *EPB41*, along with spectrin and actin, forms the RBC membrane cytoskeletal network, crucial for maintaining cell shape and deformability. Variations in the *EPB41* exon cause hereditary elliptocytosis [26]. Given the shared hematopoietic stem cell lineage of RBCs and megakaryocytes, it is plausible that *EPB41* variations may impact both RBCs and megakaryocytes. In our study, β-thalassemia patients either anemic or transfusion-dependent, did not display significant differences in multiple RBC parameters between wild-type and mutant rs78326374 of *EPB41*, suggesting limited effects on RBCs. *PPP2R5C*, newly identified in our study as associated with PLT, encodes a regulatory subunit of protein phosphatase 2A (PP2A), a major serine/threonine phosphatase modulating the phosphorylation status of numerous proteins. PPP2R5C interacts with IER*3* [27], and the deletion of *IER3* can lead to platelet and RBC defects, accompanied by thrombocytopenia [28]. Overexpression of *PPP2R5C* is observed in various leukemias, implicating its involvement in malignant transformation [29]. Leukemia patients commonly experience cytopenias across all three blood cell lineages, including a decrease in PLT. We discovered a series of missense rare variants in *TSSK1B* associated with MPV. *TSSK1B* encodes testis-specific serine kinase 1B, a member of the serine/threonine kinase family highly expressed in the testes. It is responsible for the phosphorylation and dephosphorylation events that regulate cellular signaling both intracellularly and extracellularly [30]. Further analysis of rare coding variants in *TSSK1B* revealed a statistically significant difference in these mutations among both the male patients (STAAR-O *P* = 4.42E−05) and female patients (STAAR-O *P* = 9.30E−04) (Table S5). *RNF144B*, located on the mitochondrial membrane, possesses ubiquitin–protein ligase activity and negatively regulates the processes of apoptosis and ubiquitin-dependent protein degradation. RNF144B acts as an oncogenic protein in tumor development and plays a crucial role in cell proliferation [31], which might affect platelet generation by promoting the proliferation of bone marrow megakaryocytes. Due to the specialized nature of our cohort, which focuses on a specific monogenic hematological disease, we do not currently have access to large validation cohorts for the same condition. Validating our findings through independent cohorts or functional experiments is an important direction for future research.

Our functional enrichment analyses revealed several terms closely related to platelet function, including phagocytosis, chemotaxis, platelet α granules, and adhesion. Platelets, similar to RBCs, circulate within the bloodstream and do not traverse lymphatic vessels. They primarily interact with leukocytes in organs, thereby driving and regulating host inflammatory responses and immune reactions [32]. The consistency between our analysis results and established platelet functions [33] further substantiates the credibility of our novel associated findings. Moreover, we assessed the correlation between platelet traits and six leukocyte parameters, and the results demonstrated a positive correlation between PLT and leukocyte parameters, alongside a negative correlation between MPV and leukocyte parameters. These results are consistent with our functional enrichment analyses and underscore the intricate interplay between platelets and leukocytes in modulating immune responses. Due to long-term blood transfusions and iron overload, thalassemia patients frequently experience chronic inflammation. This persistent inflammation can lead to continuous platelet activation, which results in the release of pro-inflammatory cytokines and chemokines that further aggravate the inflammatory response. Managing inflammation and controlling platelet activation through antioxidants, anti-inflammatory drugs, and antiplatelet medications can help alleviate complications, extend the patients’ lifespan, and improve their quality of life.

In conclusion, our study performed a comprehensive analysis of phenotypic heterogeneity in platelet traits among β-thalassemia patients, uncovering several novel associated genes, including *PPP2R5C*, *TSSK1B*, and *RNF144B*, previously unrecognized in the context of platelet traits. Further investigation into clinical implications, underlying mechanisms, and functional roles of these identified genes in platelet biology holds promise for enhancing our understanding and identifying potential treatment targets for platelet-related disorders. In addition, platelets are essential for thrombosis, and it is of future interest to evaluate the relationship between platelet traits and thrombotic complications in β-thalassemia patients.

## Materials and methods

### Patient recruitment

The inclusion criteria for participant recruitment were meticulously defined as follows: (1) confirmed diagnosis of β-thalassemia in patients; (2) age of 3 years or older; (3) a minimum interval of 15 days between attendance for physical examination and the last blood transfusion; and (4) absence of thalidomide intake in recent 2–3 months. A total of 1020 β-thalassemia patients were recruited from 15 medical centers spanning 13 cities in southern China. All patients received transfusion with leukocyte-depleted RBCs. Detailed medical information was systematically collected for each participant.

### Hematological analysis

Peripheral blood samples were obtained from each subject and anticoagulated with ethylenediaminetetraacetic acid-K2 (EDTA-K2). Approximately 2 ml of these samples were subjected to analysis of blood cell parameters using the BC-6000 Plus Automatic Hematology Analyzer (Mindray, Shenzhen, China).

### WGS, variant calling, and quality control

Genomic DNA was extracted from peripheral blood samples using a genomic DNA extraction kit (Catalog No. D3111-03, Magen Biotech, Guangzhou, China). WGS at an average coverage of 40× was performed on the entire cohort of 1020 patients using the DNA nanoball sequencing (DNBSEQ) platform [Beijing Genomics Institution (BGI), Shenzhen, China], and the resultant FASTQ files were provided by BGI. Subsequent quality control procedures involved filtering, alignment, sorting, marking duplicate reads to the GRCh38 reference genome, and variant calling using the Genome Analysis Toolkit (GATK) package. A total of 41.68 million variants were retained, and the information was stored in the variant calling file (VCF). These variants encompassed single nucleotide variants (SNVs) and small nucleotide insertions and deletions (InDels). Quality control criteria before GWAS encompassed gender compatibility, removal of variants with ExcessHet > 54.69, meeting a truth sensitivity filter threshold of 99.6% for SNVs and 99.0% for InDels, exclusion of variants with an inbreeding coefficient < −0.3 and long InDels (length > 50 bp), exclusion of genotypes with genotype quality (GQ) < 20 or depth (DP) < 10, elimination of variants on autosomal chromosomes with a call rate < 5%, and removal of variants violating Hardy–Weinberg equilibrium (HWE) (*P* < 1E−06). Finally, 33.43 million variants, including common variants, low-frequency variants, and rare variants, passed these quality controls and were retained for the subsequent association study. All genomic coordinates are referenced to National Center for Biotechnology Information (NCBI) GRCh38/University of California Santa Cruz (UCSC) hg38.

### Statistical analysis of clinical phenotypes

Continuous clinical data were presented as mean ± standard deviation (SD). Baseline clinical characteristics across different groups were compared using the Student’s *t*-test, Mann–Whitney U test, or Chi-squared test, as appropriate, utilizing the Statistical Package for the Social Sciences (SPSS) (v26). These statistical analyses were based on two-tailed hypothesis tests, with results considered statistically significant at *P* < 0.05. Graphical representations were generated by GraphPad Prism (v9.0) and R (v4.3.1).

### Correlation and heritability analyses of PLT and MPV

The clinical correlation was assessed by Spearman rank correlation analysis based on two-tailed hypothesis tests with a significance threshold set at *P* < 0.05. To investigate the genetic correlation between genome-wide variant data and phenotype data, the bivariate genome-based restricted maximum likelihood (GREML) [34] in the Genome-wide Complex Trait Analysis (GCTA) (v1.94.1) package [35] was employed. GCTA analyzed the PLT and MPV in the phenotype file using 8 fixed effect covariates, the first 10 principal components (PCs), and a genetic relatedness matrix (GRM). Variant-based heritability of PLT and MPV was estimated by GCTA and presented as average with corresponding SE.

### The procedure of rank-based inverse normal transformation

A two-stage procedure for rank normalization was implemented across all association studies in our research [36]. This fully adjusted two‐stage approach was chosen due to its ability to reduce excess Type I errors and improve statistical power [36]. In addition, compared to approaches without rank normalization, this approach has a lower degree of inflation [36]. To establish a “null model”, the GENESIS package (v2.20.1) [37] was utilized to construct a linear mixed model (LMM). This null model was fitted under the null hypothesis of no association between the trait and any genetic variant. The null model included 8 fixed effect covariates (*i.e.*, age, sex, the classification of *HBB* genotype, with or without *HBA* mutation, hemoglobin, transfusion-free survival time, transfusion frequency, and serum ferritin) and the first 10 PCs estimated using FastSparseGRM (v1.01) [38]. The classification of *HBB* genotype including β^0^/β^0^, β^0^/β^+^, β^0^/N, β^0^/N + α-duplication, β^+^/β^+^, and β^0^/HPFH was defined as 1 to 6. The null model also incorporated a fourth-degree sparse GRM as a random effect factor estimated using FastSparseGRM [38] to account for genetic relatedness. These PC and GRM were generated by VCF files. In stage 1, a LMM was fitted with the continuous trait of PLT or MPV as the outcome, along with 8 fixed effect covariates, PC1–PC10, and sparse GRM using the GENESIS package [37]. The resulting marginal residuals were subjected to rank-based inverse normal transformation and rescaled by their original variance. In stage 2, a second LMM was fitted using the rescaled marginal residuals as the outcome with the same 8 fixed effect covariates, PC1–PC10, and GRM in stage 1 using GENESIS [37,38].

### Annotation

Genes were assigned to each index variant by annotating with the Functional Annotation of Variants Online Resource (FAVOR) database [25] or Annotate Variation (ANNOVAR) [39] and selecting the gene with the most severe functional consequence.

### Single variant association analysis (MAF ≥ 1%)

Single variant genome-wide association analyses for the PLT and MPV traits utilized a LMM implemented in GENESIS [37], employing an additive genetic model. VCF files were converted to genomic data structure (GDS) format using SeqArray (v1.30.0) [40]. The model fitted in stage 2 facilitated score tests to interrogate the association of each variant, encompassing 8.93 million autosomal variants. The threshold for genome-wide significance was established at *P* = 5.00E−08, while suggestive significance was set at *P* = 5.00E−06. Manhattan plots and QQ plots were generated to illustrate the overall results and the significance of the association study. The genomic inflation factor (λ) was calculated to evaluate the deviation of the observed *vs.* the expected distribution of *P* values. Conditional analysis [5] was performed by conditioning on known platelet-associated variants within a 1-Mb window, with known associated variants indexed in the GWAS Catalog [21]. LocusZoom (v1.4) was employed to display the level of LD and draw regional plots [41]. LD was based on the samples included in this analysis.

### Gene-level analysis and GSEA using Multi-marker Analysis of GenoMic Annotation

The gene-level association analysis was performed on the summary statistics of single variant analysis using Multi-marker Analysis of GenoMic Annotation (MAGMA) (v1.10) [42], employing default settings [*N* (available genes) = 18,052]. Because the sample size is relatively small, we chose 0.1/*N* as a threshold for each association analysis. Bonferroni-corrected significant *P* value for the gene-level association analysis was 5.54E−06 (*P* = 0.1/18,052), and suggestive significance *P* value was 5.00E−04. Gene set-based biological and functional enrichment analyses were performed on the Gene Ontology (GO) and the Human Phenotype Ontology (HPO) [43] [*N* (available gene sets) =15,978]. The GSEA was performed to predict the impact on biological functions using the GSEA database [44]. Bonferroni-corrected significant *P* value of enrichment analysis was 6.26E−06 (*P* = 0.1/15,978).

### Low-frequency and rare variant association analyses (MAF < 5%)

STAARpipeline (v0.9.7) is a newly efficient and robust all-in-one framework for variant association detection, capable of automatically annotating WGS data and performing variant association analysis, especially for rare variants and noncoding regions of the genome [38,45]. Both gene-centric and non-gene-centric analyses are defined by categorical functional annotations [38,45]. STAARpipeline incorporated nine annotation PCs (aPCs) and three integrative scores — Combined Annotation Dependent Depletion (CADD) [46], Linear Inference of Natural Selection from Interspersed Genomically coHerent elemenTs (LINSIGHT) [47], and Functional Analysis through Hidden Markov Models with an eXtended Feature set (FATHMM-XF) [48] — as weights for constructing variant-set test for association using annotation information (STAAR) statistics. The aPCs are calculated using the first PC of the set of individual functional annotation scores measuring similar biological functionality. For coding region, STAARpipeline defined five different aggregate masks of rare variants: (1) putative loss-of-function (plof), (2) putative loss-of-function or disruptive missense (plof-Ds), (3) missense, (4) disruptive missense, and (5) synonymous. In addition, we performed an analysis across protein-coding genes, including two masks of PTV and PTV + D. Note that we did not incorporate the 12 quantitative functional annotations in STAAR statistics as in other analyses for the analyses of these two masks. For noncoding regions, STAARpipeline used seven rare variant masks: (1) promoter variants within cap analysis of gene expression (CAGE) sites (promoter–CAGE), (2) promoter variants within DHSs (promoter–DHS), (3) enhancer variants within CAGE sites (enhancer–CAGE), (4) enhancer variants within DHSs (enhancer–DHS), (5) untranslated regions (UTRs; rare variants in 3′ UTR and 5′ UTR), (6) upstream variants, and (7) downstream variants. Detailed explanations of the regions defined based on these masks are discussed within the STAARpipeline. Following the STAARpipeline tutorial, VCF files were converted to GDS format using SeqArray [40] and annotated by FAVORannotator [25] as annotated GDS (aGDS) files for rare variant analyses. The null model in stage 2 using GENESIS was converted to the STAAR null model using the STAARpipeline. More details of the analysis method can be found in [38]. For both gene-centric analysis of the coding and noncoding genomes and non-gene-centric analysis of sliding windows, *P* value of each variant set was calculated by STAAR-O, an omnibus test aggregating multiple annotation-weighted variant set tests in the STAAR framework [38,45]. For gene-centric coding analysis and gene-centric noncoding analysis of PLT and MPV, Bonferroni-corrected STAAR-O *P* = 5.00E−06 (alpha = 0.1/20,000) was considered as the genome-wide significant threshold (number of the protein-coding genes was 20,000). STAAR-O *P* = 1.00E−05 was considered as the suggestive threshold. The fixed length of the sliding window was set as 4 kb with a skip length of 2 kb. For the 4-kb sliding window analysis of PLT and MPV, Bonferroni-corrected STAAR-O *P* = 3.91E−08 (alpha = 0.1/number of windows) was considered as the genome-wide significant threshold (number of windows in PLT test was 2,559,559 and in MPV test was 2,559,381) and STAAR-O *P* = 1.00E−05 was considered as the suggestive threshold. Conditional analysis [38] was performed to identify rare variant associations independent of known platelet-associated variants, and the known associated variants were indexed in the GWAS Catalog [21]. Details of conditional analysis can be found in [38].

## Ethical statement

This study was approved by the Medical Ethics Committee of Nanfang Hospital, China (Approval No. NFEC-2019-039) following the Declaration of Helsinki. Written informed consent was obtained from all participants, either directly from those aged ≥ 18 years or from legal guardians of minors.

## Code availability

The scripts used to generate the results in this study have been submitted to BioCode at the National Genomics Data Center (NGDC), China National Center for Bioinformation (CNCB) (BioCode: BT007539), which are publicly accessible at https://ngdc.cncb.ac.cn/biocode/tool/BT007539. The scripts are also available on GitHub (https://github.com/wangshuang2024/platelet_trait_association_study).

## Supplementary Material

qzae065_Supplementary_Data

## Data Availability

All final data supporting the findings of this study have been deposited in the GWAS Atlas database [49–51] at the NGDC, CNCB (GWAS Atlas: GVP000037), and are publicly accessible at https://ngdc.cncb.ac.cn/gwas/.

## CRediT author statement

**Xingmin Wang:** Data curation, Formal analysis, Investigation, Methodology, Resources, Software, Validation, Visualization, Writing – original draft. **Qianqian Zhang:** Data curation, Investigation, Methodology, Resources, Validation, Writing – review & editing, Funding acquisition. **Xianming Chen:** Data curation, Formal analysis, Visualization, Software, Resources. **Yushan Huang:** Resources, Software. **Wei Zhang:** Data curation, Validation, Resources. **Liuhua Liao:** Resources, Funding acquisition. **Xinhua Zhang:** Resources. **Binbin Huang:** Resources. **Yueyan Huang:** Resources. **Yuhua Ye:** Data curation, Resources. **Mengyang Song:** Data curation, Resources. **Jinquan Lao:** Resources. **Juanjuan Chen:** Resources. **Xiaoqin Feng:** Resources. **Xingjiang Long:** Resources. **Zhixiang Liu:** Resources. **Weijian Zhu:** Resources. **Lian Yu:** Resources. **Chengwu Fan:** Resources. **Deguo Tang:** Resources. **Tianyu Zhong:** Resources. **Mingyan Fang:** Resources, Software. **Caiyun Li:** Resources. **Chao Niu:** Data curation, Resources. **Li Huang:** Data curation, Resources. **Bin Lin:** Resources. **Xiaoyun Hua:** Resources. **Xin Jin:** Resources, Software. **Zilin Li:** Conceptualization, Project administration, Supervision, Writing – review & editing. **Xiangmin Xu:** Conceptualization, Funding acquisition, Project administration, Resources, Supervision, Writing – review & editing. All authors have read and approved the final manuscript.

## ORCID

0000-0002-0839-5647 (Xingmin Wang)

0000-0003-1624-036X (Qianqian Zhang)

0009-0004-2151-2328 (Xianming Chen)

0000-0001-8700-620X (Yushan Huang)

0009-0006-1586-8955 (Wei Zhang)

0000-0002-2869-3705 (Liuhua Liao)

0000-0003-4470-2410 (Xinhua Zhang)

0009-0000-6236-393X (Binbin Huang)

0000-0003-4489-4942 (Yueyan Huang)

0000-0002-8037-2175 (Yuhua Ye)

0009-0005-7034-8824 (Mengyang Song)

0009-0002-0796-9538 (Jinquan Lao)

0009-0006-6267-3536 (Juanjuan Chen)

0000-0003-1166-0138 (Xiaoqin Feng)

0009-0004-6530-5486 (Xingjiang Long)

0009-0009-7038-8905 (Zhixiang Liu)

0000-0002-2120-5860 (Weijian Zhu)

0009-0005-1807-3827 (Lian Yu)

0009-0002-1549-5037 (Chengwu Fan)

0009-0002-0433-0712 (Deguo Tang)

0000-0003-1934-0032 (Tianyu Zhong)

0000-0001-7185-6445 (Mingyan Fang)

0009-0002-2708-3793 (Caiyun Li)

0009-0007-1302-9127 (Chao Niu)

0009-0009-1320-0996 (Li Huang)

0009-0004-4089-9951 (Bin Lin)

0009-0002-3884-2388 (Xiaoyun Hua)

0000-0001-7554-4975 (Xin Jin)

0000-0003-1521-8945 (Zilin Li)

0000-0002-8652-1194 (Xiangmin Xu)
