# Supplementary material for: Whole-genome Sequencing Association Analysis of Quantitative Platelet Traits in A Large Cohort of β-thalassemia
Source: Genomics Proteomics Bioinformatics. 2024 Sep 27;23(2):qzae065. doi: 10.1093/gpbjnl/qzae065 (PMC12373638; doi:10.1093/gpbjnl/qzae065)
Supplement: qzae065_Supplementary_Data [file qzae065_supplementary_data.zip › Table S5-done.docx]

| Table S5 Gene-centric coding analysis results of TSSK1B for MPV analysis in male and female subgroup | | | | | | |
| --- | --- | --- | --- | --- | --- | --- |
| **Type** | **Trait** | **Gene** | **Chr** | **Category** | **SNV** | **STAAR-O (unconditional)** |
| Female | MPV | *TSSK1B* | 5 | Missense | 6 | 9.30E**−**04 |
| Male | MPV | *TSSK1B* | 5 | Missense | 10 | 4.42E**−**05 |
|  | | | | | | |
