# Supplementary material for: Whole-genome Sequencing Association Analysis of Quantitative Platelet Traits in A Large Cohort of β-thalassemia
Source: Genomics Proteomics Bioinformatics. 2024 Sep 27;23(2):qzae065. doi: 10.1093/gpbjnl/qzae065 (PMC12373638; doi:10.1093/gpbjnl/qzae065)
Supplement: qzae065_Supplementary_Data [file qzae065_supplementary_data.zip › supplementary material captions.docx]

**Supplementary materials**

**Figure S1 The detail information of top variants in single variant association analyses**

**A.** The regional plot of association results for rs78326374 (C>A). The top variant is marked by a purple diamond. The color of each point indicates the linkage disequilibrium r^2^ with top variant. **B.** The regional plot of association results for rs144319949 (C>A). **C.** The regional plot of association results for rs117379094 (T>C). **D**. Statistical analysis of PLT compared in homozygous or heterozygous patients with wild-type patients of rs78326374. **E.** Statistical analysis of PLT compared in heterozygous patients with wild-type patients of rs144319949. **F.** Statistical analysis of MPV compared in heterozygous patients with wild-type patients of rs117379094. Ns, *P* > 0.05; *, *P* ≤ 0.05; **, *P* ≤ 0.01; ***, *P* ≤ 0.001; ****, *P* ≤ 0.0001. The data of Figure S1 D–F were showed in Table S6.

**Figure S2 The relationship between platelet traits and inflammatory cells**

**A.** The relationship between PLT and WBC count, showing a positive relationship. **B.** The relationship between PLT and monocyte count. **C.** The relationship between PLT and lymphocyte count. **D.** The relationship between PLT and neutrophil count. **E.** The relationship between PLT and basophil count. **F.** The relationship between PLT and eosinophil count. **G.** The relationship between MPV and WBC count, showing a negative relationship. **H.** The relationship between MPV and monocyte count. **I.** The relationship between MPV and lymphocyte count. **J.** The relationship between MPV and neutrophil count. **K.** The relationship between MPV and basophil count. **L.** The relationship between MPV and eosinophil count. WBC, white blood cell.

**Figure S3 The results of the PTV association study for PLT and MPV using STAARpipeline**

**A.** Manhattan plot for unconditional PLT gene-centric coding analysis of PTVs and PTVs + D. **B.** QQ plot for unconditional PLT gene-centric coding analysis of PTVs and PTVs + D. **C.** Manhattan plot for unconditional MPV gene-centric coding analysis of PTV and PTV + D. **D.** QQ plot for unconditional MPV gene-centric coding analysis of PTVs and PTVs + D. PTV, protein-truncating variant; PTV + D, protein-truncating + disruptive missense variant.

**Table S1 The suggestive significant variants in single variant association study of PLT and MPV (*P* < 5.00E−06)**

**Table S2 Known variants list of PLT and MPV in ploting QQ plot and conditional analysis**

**Table S3 The results of significant and suggestive genes in gene-based association study of PLT and MPV in β-thalassemia cohort**

**Table S4 The top 50 items in gene set-based enrichment test of PLT and MPV in β-thalassemia cohort**

**Table S5 Gene-centric coding analysis results of *TSSK1B* for MPV analysis in male and female subgroup**

**Table S6 The phenotype of platelet trait with homozygous or heterozygous patients of rs78326374 (C>A), rs144319949 (C>A), and** **rs117379094 (T>C)**
