# Supplementary figures and images for: Whole-genome Sequencing Association Analysis of Quantitative Platelet Traits in A Large Cohort of β-thalassemia
Source: Genomics Proteomics Bioinformatics. 2024 Sep 27;23(2):qzae065. doi: 10.1093/gpbjnl/qzae065 (PMC12373638; doi:10.1093/gpbjnl/qzae065)

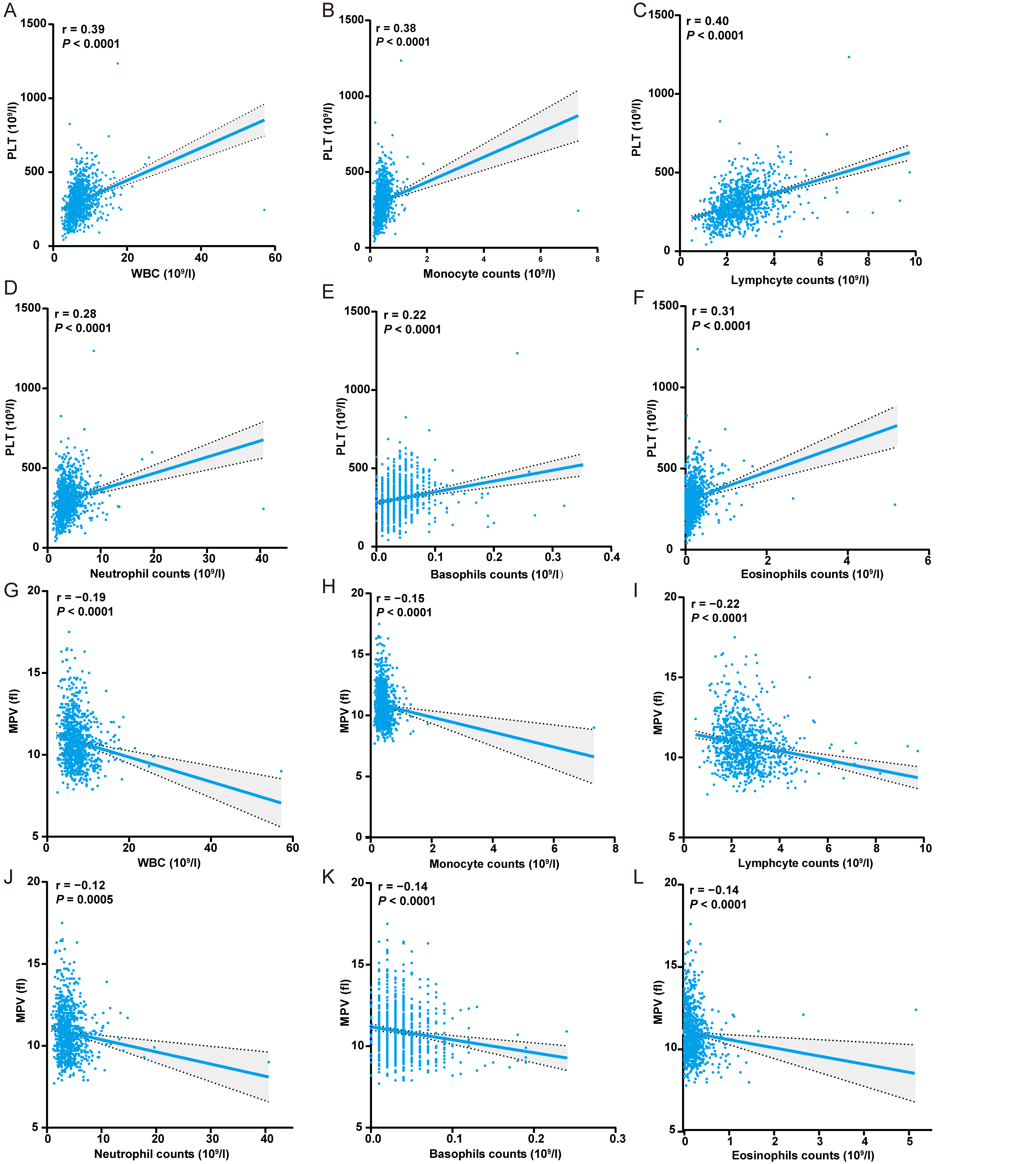

Supplement: qzae065_Supplementary_Data [file qzae065_supplementary_data.zip › figure S2.tif]

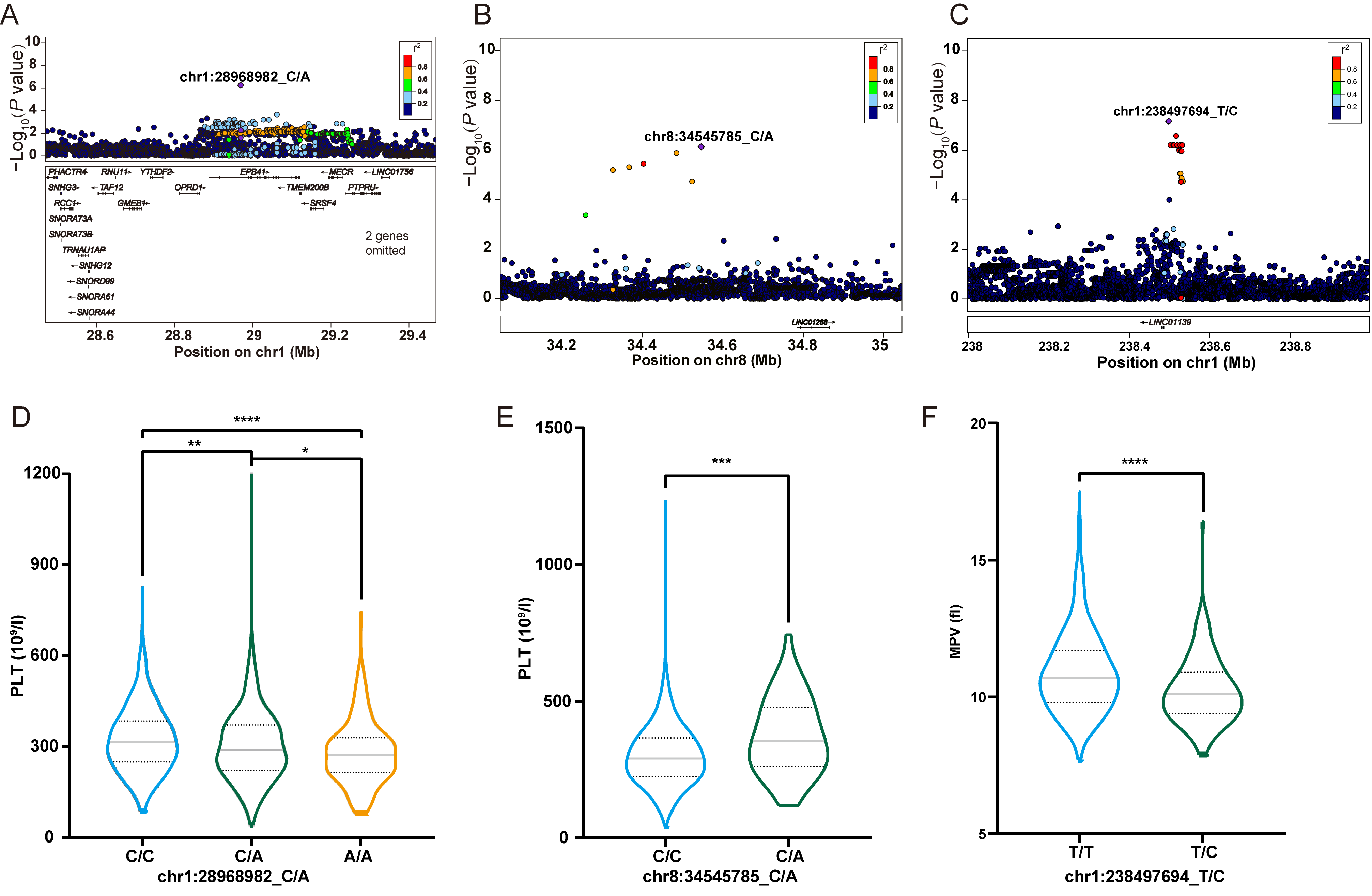

Supplement: qzae065_Supplementary_Data [file qzae065_supplementary_data.zip › Figure S1.tif]

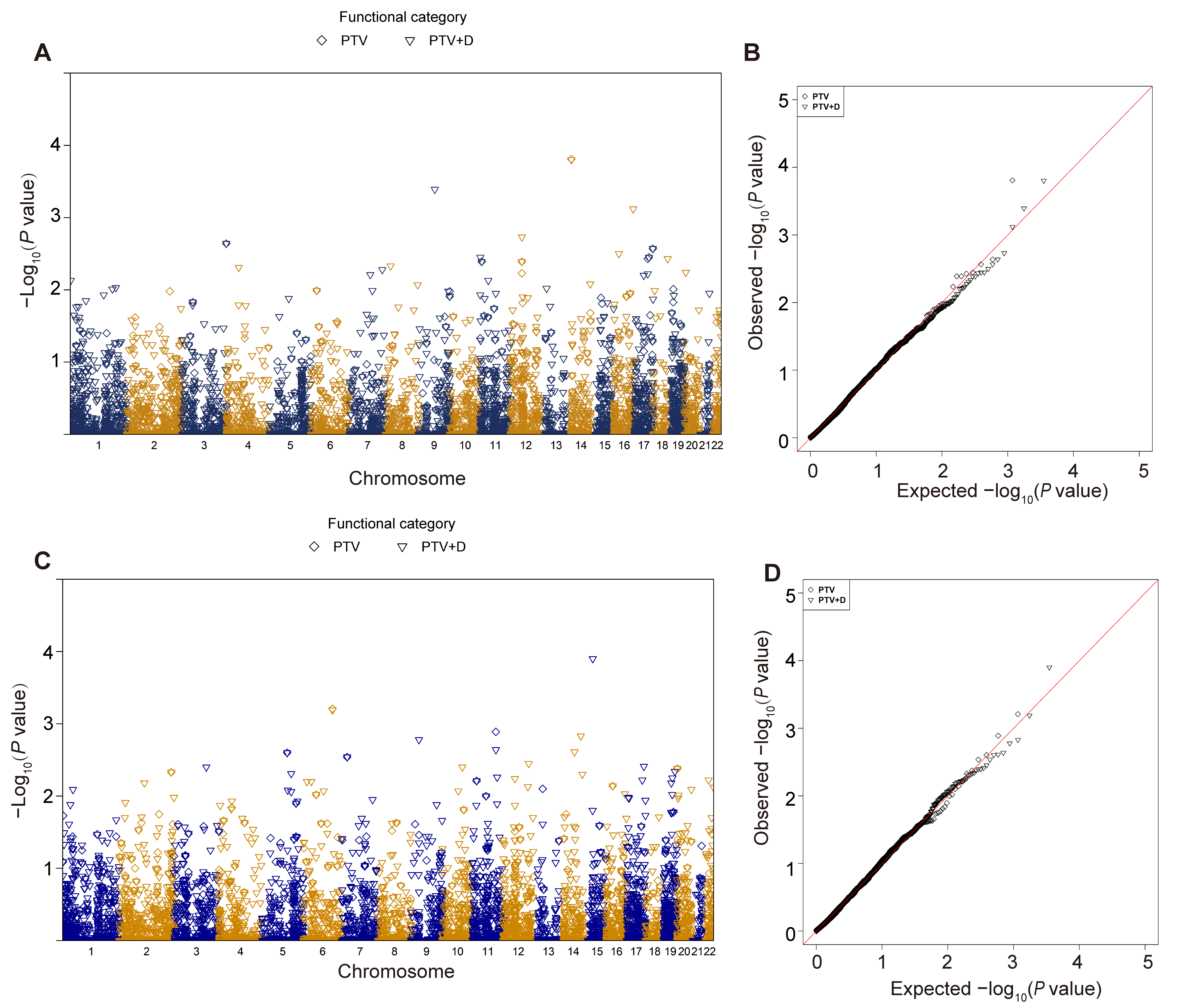

Supplement: qzae065_Supplementary_Data [file qzae065_supplementary_data.zip › Figure S3.tif]
